# Development of a membrane-anchored ligand and receptor yeast two-hybrid system for ligand-receptor interaction identification

**DOI:** 10.1038/srep35631

**Published:** 2016-10-20

**Authors:** Jingjing Li, Jin Gao, Lei Han, Yinjie Zhang, Wen Guan, Liang Zhou, Yan Yu, Wei Han

**Affiliations:** 1 Laboratory of Regeneromics, School of Pharmacy, Shanghai Jiao Tong University, Shanghai, China; 2 Shanghai Municipality Key Laboratory of Veterinary Biotechnology, School of Agriculture and Biology, Shanghai Jiao Tong University, Shanghai, China

## Abstract

Identifying interactions between ligands and transmembrane receptors is crucial for understanding the endocrine system. However, the present approaches for this purpose are still not capable of high-throughput screening. In this report, a membrane-anchored ligand and receptor yeast two-hybrid (MALAR-Y2H) system was established. In the method, an extracellular ligand is linked with an intracellular split-ubiquitin reporter system via a chimeric transmembrane structure. Meanwhile, the prey proteins of transmembrane receptors are fused to the other half of the split-ubiquitin reporter system. The extracellular interaction of ligands and receptors can lead to the functional recovery of the ubiquitin reporter system in yeast, and eventually lead to the expression of report genes. Consequently, the system can be used to detect the interactions between extracellular ligands and their transmembrane receptors. To test the efficiency and universality of the method, interactions between several pairs of ligands and receptors of mouse were analyzed. The detecting results were shown to be thoroughly consistent with the present knowledge, indicating MALAR-Y2H can be utilized for such purpose with high precision, high efficiency and strong universality. The characteristics of the simple procedure and high-throughput potential make MALAR-Y2H a powerful platform to study protein-protein interaction networks between secreted proteins and transmembrane proteins.

Identifying interactions between cytokines and transmembrane receptors is important for understanding the endocrine regulation system and drug development. Since receptors are accessible to drugs, many important cytokine pathways, such as PD1 and VEGF/EGFR, are drug targets. However, there are still many unknown interactions that need to be mined. Defining these interactions will absolutely aid us in understanding the interaction networks involving cytokines and transmembrane receptors better and will accelerate drug development.

If the nervous system is a “tele-communications network”, the cytokine-receptor network can be seen as a “broadcasting network” between organs or cells. However, studying such a complex network is a huge challenge. Since the cellular membrane is a prerequisite for spatial construction of receptors, especially multi-transmembrane receptors, most *in vitro* methods, including surface plasmon resonance^1^, are not suitable to study these membrane-bound protein systems. Moreover, *in vivo* methods, such as the Ca^2+^ flux assay, chemotaxis assay and competitive binding assay^2^, have major drawbacks, including low-throughput and complex processes, which make these assays unsuitable for high-throughput screening.

Yeast two-hybrid (Y2H) method, as an important protein-protein interaction (PPI) assay, has contributed significantly to proteomics in the post-genomic era. Currently, variant Y2H methods have been used for detecting PPIs between two cytoplasmic proteins, between a cytoplasmic protein and a membrane protein, and between two membrane-associated proteins^3^. However, all these methods are not suitable for detecting PPIs between secreted cytokines and transmembrane receptors for two reasons: (i) almost all transmembrane proteins require the plasma membrane to form native structures; and (ii) PPIs occurring on the outer cytoplasmic membrane need to turn on an intracellular signal cascade that can be readily detected.

Previously, a membrane protein Y2H system to detect PPIs between two membrane proteins was developed^4^. In the method, the yeast ubiquitin gene is split into N- and C-terminal fragments (N_ub_ and C_ub_). The two separated fragments are fused, respectively, with the membrane-associated bait and prey proteins of interest. The interaction of bait and prey brings N_ub_ and C_ub_ together to reconstitute a functional ubiquitin. This reconstituted ubiquitin is recognized by an endogenic ubiquitin specific protease (UBP) that leads to the release of a fused transcription factor from the C-terminus of C_ub_ and the transcription of reporter genes.

Inspired by this strategy, we hypothesized that, if an extracellular ligand of interest (bait) could be linked with an intracellular reporter system, this approach could be used to detect interactions between the bait and candidate transmembrane receptors (prey). The hypothesis was finally verified through a series of experiments. Herein, in the present article, we are going to describe the successful development of the membrane-anchored ligand and receptor yeast two-hybrid (MALAR-Y2H) method to detect interactions between extracellular cytokines and transmembrane receptors.

## Results

### Experiment Design

CXC-motif chemokine ligand 12 (CXCL12), a classic chemokine, is a member of the CXC motif chemokine gene family. CXCL12 binds two known receptors, CXCR4 and CXCR7. Both receptors belong to the CXCR family, a type of seven-transmembrane domain G-protein-coupled receptors (GPCR)^5^. To verify the effectiveness of the MALAR-Y2H system, we attempted to examine the interactions between CXCL12 and its known receptors and other candidate receptors.

**Figure 1:**
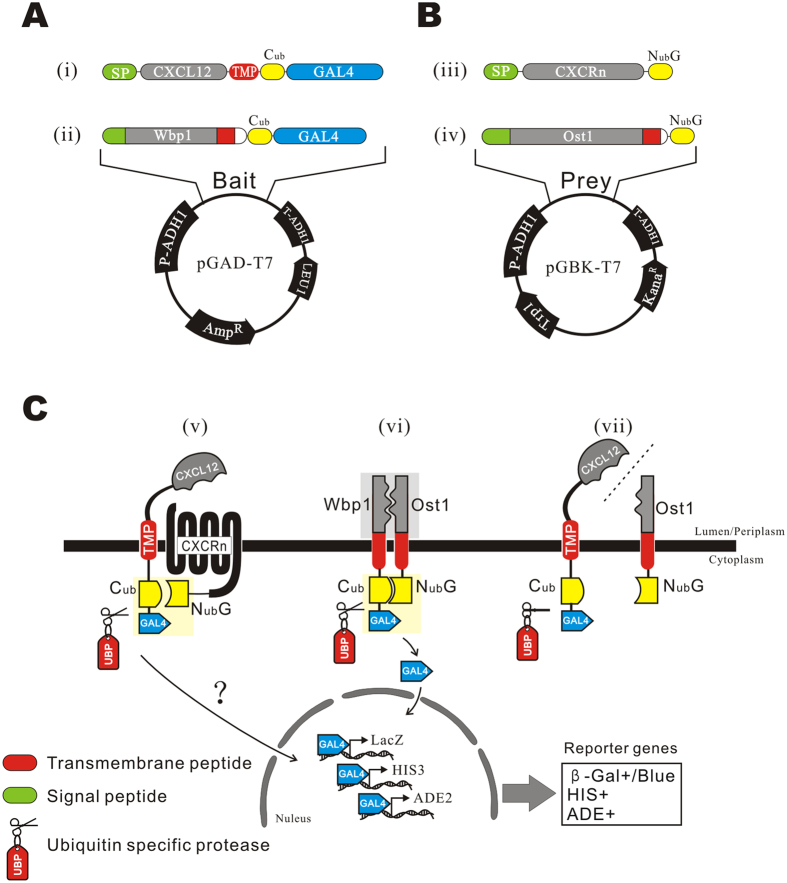
Design of bait and prey plasmids and the principles of detecting the interaction. (**A**) The structures of the bait plasmids. (i) CXCL12 was fused with a signal peptide (SP) at the N-terminus and with a transmembrane peptide (TMP) at C-terminus, followed by C_ub_ and GAL4 in tandem. (ii) WBP1 was fused with C_ub_ and GAL4 as a control. Bait genes were cloned into the plasmid pGAD-T7. (**B**) Structures of prey plasmids. (iii) The prey fusion was composed of a SP, CXCR and N_ub_*G* in tandem. (iv) OST1 was fused with N_ub_*G* as a control prey. Prey genes were cloned into the plasmid pGBK-T7. (**C**) Principles of detecting the interaction. (v) The bait protein is expressed as a transmembrane protein, in which the CXCL12 domain is directed into the lumen (and the periplasm, if mature forms are located on plasmalemma) and linked with the C_ub_-GAL4 cassette in the cytoplasm by the TMP. CXCRs-N_ub_*G* is co-expressed on the plasma membrane. The interaction of CXCL12 and CXCRs unites C_ub_ and N_ub_*G* and leads to the reconstitution of the split ubiquitin protein, which is recognized and cleaved by UBP to release GAL4. The liberated GAL4 protein is transported into the nucleus by the SV40 nuclear localization signal at its N-terminus, and transcription of reporter genes *lacZ*, *HIS3* and *ADE2* results in blue clones on a SD/His^−^Ade^−^ plate in the presence of *α*-X-Gal. (vi) Coexpression of WBP1-C_ub_-GAL4 and OST1-N_ub_*G* was used as the positive interacting proteins control. The two genes are known interacting partners of membrane proteins of yeast. (vii) Coexpression of SP-CXCL12-TMP-C_ub_-GAL4 and OST1-N_ub_*G*, two unrelated genes, was used as a negative control.

The bait protein was generated by constructing an artificial transmembrane structure (Fig. 1A,i), in which five elements were fused in tandem, including: **(1)** a signal peptide (SP), from SP of CXCL12, Wbp1 (a yeast transmembrane protein), or MFAL1 (a yeast secretory protein), **(2)** the bait protein CXCL12, **(3)** a transmembrane peptide (TMP) from yeast protein Wbp1, **(4)** a C-terminal fragment of yeast ubiquitin (C_ub_) and **(5)** a transcription factor, Regulatory Protein GAL4 (GAL4). The artificial bait protein possesses the same topological structure of Type III membrane proteins^6^.

The candidate prey proteins, CXC motif receptors, were fused with (a) an SP, from yeast gene Wbp1, Ost1 or MFAL1 respectively, at N-terminus; (b) a mutant N-terminal fragment of ubiquitin (N_ub_*G*) at the C-terminus (Fig. 1B,iii). N_ub_*G* has an Ile13Gly mutation when compared with the wild-type sequence, which inhibits spontaneous reconstitution between native N_ub_ and C_ub_^4^.

In theory, the SP can guide N-terminus of the bait protein targeting to the endoplasmic reticulum (ER) lumen and to the extracellular space. If the mature forms are located on plasmalemma, and the TMP anchors the bait protein to the ER membrane, they can conjugate with the reporter modules, C_ub_ and GAL4, which exist in the cytoplasm (Fig. 1A,i)^6^. Therefore, preys will be directed to integrate with the ER membrane with a N_ub_ fragment in the cytoplasm (Fig. 1B,iii).

Interaction of SP-CXCL12-TMP-C_ub_-GAL4 and SP-CXCR-N_ub_*G* reconstructs the split C_ub_ and N_ub_*G* into a whole and activated ubiquitin, which can be recognized by ubiquitin-specific protease (UBP) and leads to the release of GAL4 from the C-terminus of C_ub_ (Fig. 1C,v).

Co-expression of WBP1-C_ub_-GAL4 and OST1-N_ub_*G* was used as a positive control (Fig. 1A,ii,B,iv). WBP1 and OST1 are transmembrane proteins of *S. cerevisiae*, and are interacting subunits of the N-oligosaccharyl transferase (OST) complex. Their co-expression leads to the physical reconstitution and functional recovery of ubiquitin via colocalizing C_ub_ and N_ub_*G* (Fig. 1C,vi). Co-expression of SP-CXCL12-TMP-C_ub_-GAL4 and OST1-N_ub_*G* was used as a negative control (Fig. 1C,vii).

### Subcellular Localization of Baits and Preys

**Figure 2:**
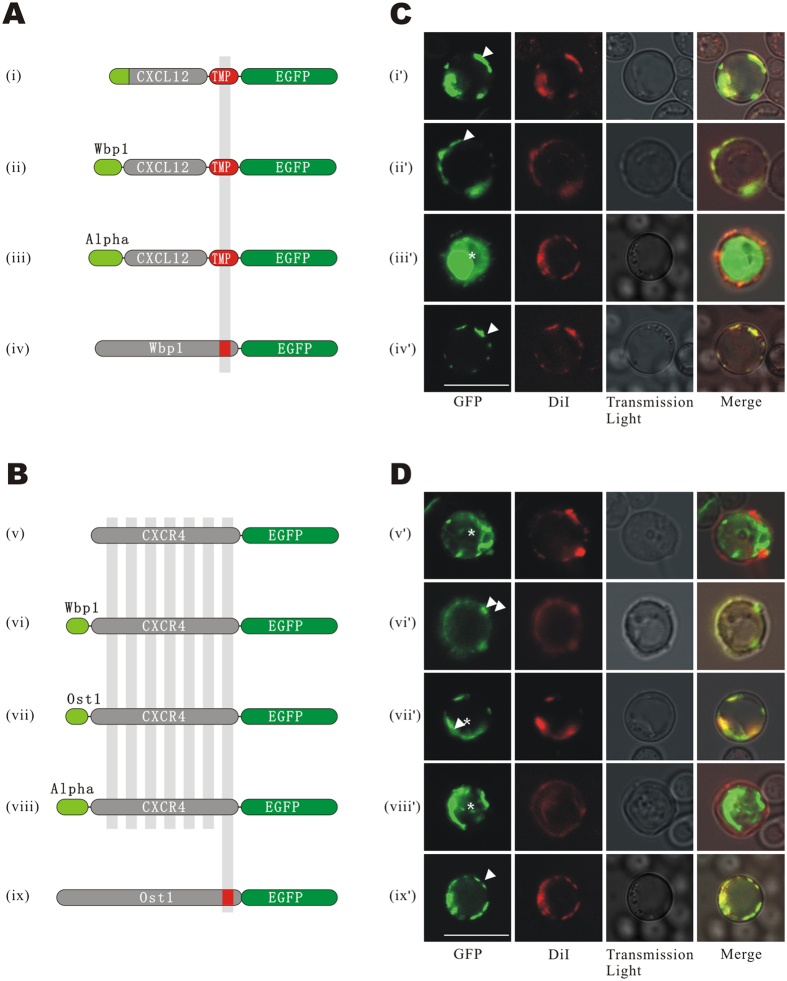
Subcellular localization of baits and preys with different signal peptides. (**A**) Structure of bait plasmids. The pGAD-T7 vector was used as the backbone of the bait plasmids, in which bait genes were promoted by the ADH1 promotor. Three different signal peptides, (i) SP_CXCL12_, (ii) SP_WBP1_, or (iii) SP_MFAL1_ were fused at the N-terminal end of CXCL12, and the transmembrane peptide of TMP_WBP1_ and EGFP were fused to the C-terminus of CXCL12 in tandem. (iv) WBP1-EGFP was used as the positive control. (**B**) Structures of prey plasmids. The pGBK-T7 vector was used as the backbone of the prey plasmids, in which preys were promoted by the ADH1 promoter. (v) CXCR4 with the native N-terminus or CXCR4 fused with different signal peptides, (vi) SP_WBP1_, (vii) SP_OST1_, or (viii) SP_MFAL1_ were tagged with EGFP at the C-terminus. (ix) OST1-EGFP was used as the control. (**C,D**) LSCM images of EGFP. Baits (i’-iv’) and preys (v’-ix’) were expressed by GoldY2H and Y187, respectively, and observed with LSCM. Images of EGFP and membrane-specific fluorescent dye DiI and transmission light were captured and merged. The bars indicate 10 micrometers. The arrows direct the membrane localization of baits and preys, and the asterisks indicate the unexpected intracellular localizations.

To test the secretory efficacy of different signal peptides, we tried signal peptides derived from three origins, including SP_CXCL12_ (signal peptide of CXCL12, residues 1–28) (Fig. 2A,i), SP_WBP1_ (*S. cerevisiae* WBP1, residues 1–31) (Fig. 2A,ii) and SP_MFAL1_ (*S. cerevisiae* MFAL1, residues 1–89) (Fig. 2A,iii), as the signal peptide for the bait protein. The transmembrane peptide TMP_WBP1_ (WBP1, residues 350–430) was used as the TMP. Enhanced green fluorescence protein (EGFP) was in-frame fused at the C-terminus of the TMP to visualize protein location. WBP1-EGFP was used as positive control (Fig. 2A,iv).

Bait plasmids were transformed into GoldY2H host cells and the co-localization of bait protein and plasma membrane was observed. The fluorescence of EGFP-tagged bait protein and DiI-stained plasma membrane were captured using laser scanning confocal microscopy (LSCM). The result showed that green fluorescence in host cells of SP_CXCL12_-CXCL12-TMP_WBP1_-EGFP and SP_WBP1_-CXCL12-TMP_WBP1_-EGFP focused accurately to the plasma membrane (Fig. 2C,i’–ii’). In contrast, the fluorescent signal of SP_MFAL1_-CXCL12-TMP_WBP1_-EGFP expressing cells was distributed intracellularly (Fig. 2C,iii’). Thus, it was indicated that SP_CXCL12_ and SP_WBP1_ guided bait secretion and anchoring to the cell plasma membrane correctly rather than SP_MFAL1_. The positive control protein WBP1-EGFP was found to locate on the plasma membrane, as expected (Fig. 2C,iv’).

CXCR4, a well-known CXCL12 receptor, was used as a representative to optimize the localization of prey. Signal peptides SP_WBP1_, SP_OST1_ (yeast OST1, residues 1–30), or SP_MFAL1_ were fused at the N-terminus of CXCR4 (Fig. 2B,v–viii). EGFP was fused at the C-terminus as a reporter gene for subcellular localization imaging (Fig. 2B,ix).

Observations revealed that CXCR4 was directed onto the plasma membrane by both SP_WBP1_ and SP_OST1_ (Fig. 2D,vi’,vii’), but not by SP_MFAL1_ or its native N-terminus (Fig. 2D,v’,viii’). The fluorescent signals of the latter two preys were distributed mainly in the cytoplasm or vacuole. The positive control OST1-EGFP was well co-localized with DiI- stained plasma membrane (Fig. 2D,ix’).

Based on the above results, SP_CXCL12_ and SP_OST1_ were selected as the optimal signal peptides to direct localization of the baits and preys respectively in the following experiments. The localization assays of other CXCRs are shown in Figure S1.

#### Construction of Y2H Plasmids

We then introduced the split ubiquitin system to bait and prey proteins to generate the reporter module. The split ubiquitin system was composed of two parts. Part I, containing the C_ub_ fragment and transcript factor GAL4 in tandem, was fused at the C-terminal to the TMP of the bait. Part II, the N_ub_*G* fragment was fused to the 3′ end of prey genes.

#### Self-Activation Detection

**Figure 3:**
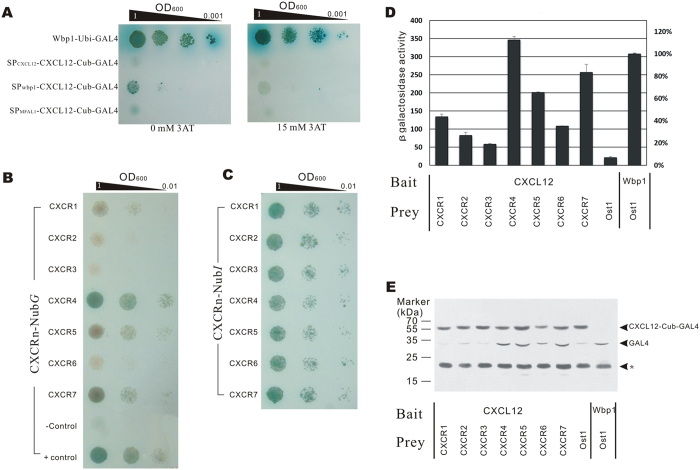
PPIs of the CXCL12 and CXCR family. (**A**) Self-activation detection. GoldY2H cells expressing bait protein with SP_CXCL12_, SP_WBP1_ and SP_MFAL1_ signal peptides were detected to self-activate. Five microliter diluted suspensions were dropped onto SD/Trp^−^Ade^−^His^−^ plates with *α*-X-gal and 3-AT, and grown for 72 h. (**B**) Cells growth assay. GoldY2H cells expressing the SP_CXCL12_-CXCL12-TMP-C_ub_-GAL4 plasmid was hybridized with Y187 cells expressing each SP_OST1_-CXCR-N_ub_*G* fusion protein, and diploid cells were dropped onto SD/Leu^−^Trp^−^Ade^−^His^−^ plates containing *α*-X-gal and 15 mM 3-AT. The growth of cells was observed after 72 h cultivation. SP_CXCL12_-CXCL12-TMP-C_ub_-GAL4 × OST1-N_ub_*G* were used as a negative control, and WBP1-C_ub_-GAL4 × OST1-N_ub_*G* were used as a positive control. (**C**) Growth assay of cells expressing SP_CXCL12_-CXCL12-TMP-C_ub_-GAL4 and CXCR-N_ub_I. (**D**) Quantitative β-Gal assay of diploid cells expressing SP-CXCL12-TMP-Cub-GAL4 and each of the SP_OST1_-CXCR-N_ub_*G* proteins. The experiment was repeated three times independently and every sample was tested twice in each experiment (*n* = 2). (**E**) Western blot analysis of cleaved GAL4. Diploid cells expressing SP_CXCL12_-CXCL12-TMP-C_ub_-GAL4 and each of the CXCR-N_ub_*G* proteins were lysed and the GAL4 level detected by western blot analysis. The molecular weight (MW) of SP_CXCL12_-CXCL12-TMP-C_ub_-GAL4 is 59 kDa, and the MW of cleaved GAL4 is 31 kDa. The experiment was performed three times independently. The asterisk denotes a non-specific band.

Before detecting the PPIs between baits and preys, we first examined whether there was any self-activation of baits. CXCL12 bait, guided by three different signal peptides, SP_CXCL12_, SP_wbp1_ and SP _MFAL1_ respectively, were expressed in GoldY2H yeast and inoculated on selective plates with *α*-X-gal. The results indicated that baits with different signal peptides have variant degrees of self-activation, reflecting the existence of UBP-independent release of GAL4. The bait with SP_CXCL12_ showed lower self-activation (Fig. 3A,i). By adding 15mM 3-AT, a competitive inhibitor of the His3 reporter gene, into the selective medium, the self-activation was mostly inhibited (Fig. 3A,ii).

Consequently, SP_CXCL12_ was used as bait signal peptide and 15 mM 3-AT was supplemented in yeast medium in all the following tests.

### PPI Detection of CXCL12 and the CXCR Family

#### Growth Assay

GoldY2H cells harboring bait plasmids and Y187 cells harboring prey plasmids were hybridized and selected on SD/Leu^−^Trp^−^ plates. Hybrids were spread on SD/Leu^−^Trp^−^His^−^Ade^−^ medium supplemented with *α*-X-gal. The colors of colonies showed that CXCL12 can interact with CXCRs with different interaction intensities, of which CXCR4 is the strongest interaction partner. CXCR7-CXCL12 and CXCR5-CXCL12 were also found able to interact, but with a lower intensity than CXCR4-CXCL12. CXCR3-CXCL12 and CXCR6-CXCL12 were found to be the weakest interaction partners (Fig. 3B).

#### System Verification

To exclude bias caused by difference in the expression levels of prey proteins, we substituted the N_ub_*G* in the prey protein of a wild-type ubiquitin N-terminus, N_ub_*I*, which could associate with C_ub_ spontaneously and lead to the release of GAL4 independent of the interaction of bait and prey. Therefore, the strength of the reporter genes was dependent on the expression level of the prey protein rather than the interaction between the bait and prey. The result revealed that cells expressing different CXCRs grew at a similar rate, suggesting similar expression levels between preys (Fig. 3C).

#### β-Galactosidase (β-Gal) Activity Assay

We further tested the β-Gal activity of each hybrid cell. The results were consistent with the cell growth test. β-Galactosidase activity in the yeast cells expressing CXCR4-N_ub_*G*, CXCR7-N_ub_*G*, or CXCR5-N_ub_*G* were determined to be 113%, 83% and 65% β-Gal activity compared to the positive control (Fig. 3D).

#### Western Blot Assay

GAL4 cleavage caused by bait and prey interactions were detected by a western blot assay. The results revealed that GAL4 released from the bait fusion protein is induced mainly by coexpression of CXCR4-, CXCR5- and CXCR7-N_ub_*G*. This observation indicates that these three receptors can strongly interact with CXCL12 and the result is coincident with the growth assay and *β*-Gal activity assay (Fig. 3E).

### PPIs Detection between CXCL12 and CXCR5 Motifs

Based on the above experiments, we have confirmed the PPIs between CXCL12 and its functional receptor CXCR4, CXCR7 and these two receptors are known to be the of CXCL12^7^,^8^. CXCR5 was also shown to be capable of strongly interacting with CXCL12, which has not been reported so far.

**Figure 4:**
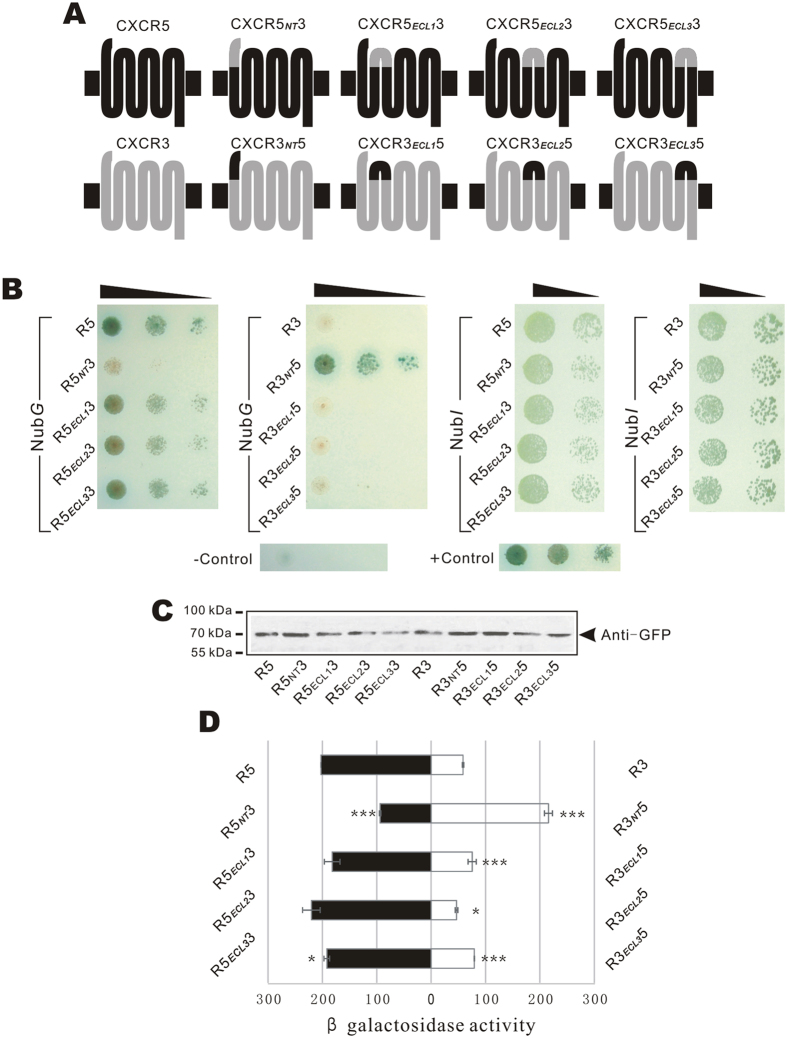
Detection of PPIs between CXCL12 and chimeric CXCR5. (**A**) Structures of chimeric receptors of CXCR5 and CXCR3. Four extracellular fragments of CXCR5 were exchanged with counterparts of CXCR3 to form eight chimeric receptors. (**B**) Cell growth detection. Diploid cells expressing SP_CXCL12_-CXCL12-TMP-C_ub_-GAL4 and CXCR5-N_ub_*G*, CXCR3-N_ub_*G*, or N_ub_*G* tagged chimeric receptors were detected by the growth ability on selection plate (SD-Leu^−^Trp^−^Ade^−^His^−^) with 15 mM 3AT + α-X-gal (left column). Diploid cells harboring the bait and N_ub_*I* tagged preys were detected (right column). Positive and negative control cells were the same as Fig. 3B. (**C**) Expression detection by western blot assay. The chimeric preys labeled with EGFP were detected by western blot. (**D**) Quantitative β-Gal assay of diploid cells expressing SP_CXCL12_-CXCL12-TMP-C_ub_-GAL4 and N_ub_*G* tagged chimeric receptors. The experiment was repeated three times independently and every sample was tested twice in each experiment (*n* = 2). * and *** indicate significance *p* < 0.05 and *p* < 0.001 of a t-test between chimeric receptors and wild-type counterparts.

To further understand the interacting features of CXCL12 and CXCR5, we investigated the exact extracellular interaction sites by domain substitution^9^. According to the UniProt database, four extracellular domains, including an N-terminal fragment (NT) and three extracellular loops (ECLs), were exchanged with homologous fragments of CXCR3, the receptor showing the weakest interaction with CXCL12 (Fig. 4A). The partitions of CXCR5 and CXCR3 sequences are given in Fig. S2.

Eight chimeric receptors fusing the SP_OST1_ signal peptide and N_ub_*G* tags were transformed into Y187 cells, and hybridized with GoldY2H cells expressing SP_CXCL12_-CXCL12-TMP-C_ub_-GAL4. Growth of hybrid cells was detected on nutrition selective plates with X-gal and 15 mM 3-AT. The results revealed that cells expressing bait and chimeric receptor CXCR5_NT_3-N_ub_*G* (CXCR5-N_ub_*G* with substituted N-terminus) lost growth ability. In contrast, cells with CXCR3_NT_5-N_ub_*G* gained growth ability (Fig. 4B,I,ii). Cells expressing bait and CXCR5_ECL1_3-N_ub_*G*, CXCR5_ECL2_3-N_ub_*G*, or CXCR5_ECL3_3-N_ub_*G* grew slower than cells with CXCR5-N_ub_*G*. Correspondingly, cells with CXCR3_ECL1_5-N_ub_*G*, CXCR3_ECL2_5-N_ub_*G*, or CXCR3_ECL3_5-N_ub_*G* gained stronger growth ability than their wild-type counterpart, CXCR3-N_ub_*G*. Using the β-Gal assay, we quantitatively evaluated the interactions between chimeric receptors and CXCL12 (Fig. 4D). The β-Gal activity of cells expressing CXCR5_NT_3-N_ub_*G* was 49% relative to the activity of wild-type CXCR5 (*p* < 0.0001, *n* = 2). The activity of CXCR3_NT_5-N_ub_*G* expression cells was equal to the activity of CXCR5 (106%, *p* = 0.065). The β-Gal activities of cells with CXCR5_ECL1_3-N_ub_*G* or CXCR5_ECL3_3-N_ub_*G* was 90% (*p* = 0.088) or 95% (*p* = 0.05) of CXCR5-N_ub_*G*. In contrast, CXCR3_ECL1_5 or CXCR3_ECL3_5 increased β-Gal activity in their host cells by 28% and 35% (*p* = 0.046 and 0.0014), respectively. Exchange of ECL2 did not significantly change the β-Gal activities for either CXCR5 (*p* = 0.14) or CXCR3 (*p* = 0.013).

Taken together, the result suggests that the N-terminus of CXCR5 is the main interaction motif in its PPI with CXCL12, at the same time ECL1 and ECL3 also contribute to the PPI, but to a lesser extent.

To avoid the bias caused by different prey expression level, we tested the interactions of the bait and N_ub_*I*-labeled prey proteins. The result showed that cells harboring N_ub_*I*-tagged preys had a similar growth ability, indicating an equal expression level of the preys (Fig. 4B,iii,iv). The expression level of the preys were also tested by western blot. To avoid the interruption of intrinsic ubiquitinated proteins, the preys were labeled with EGFP and blotted by anti-EGFP antibody. The result suggested that the expression level of the different chimeric preys are similar (Fig. 4C).

### Verification of the CXCL12 and CXCR5_NT_ interaction

**Figure 5:**
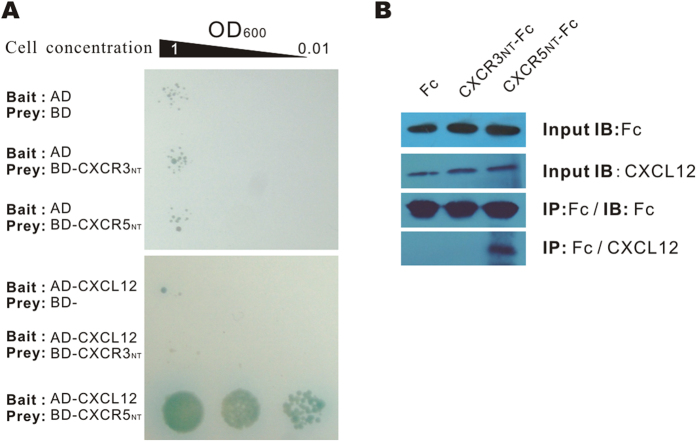
Detection of the interactions between CXCL12 and CXCR5_NT_. (**A**) The interaction between CXCL12 and the N-terminus of CXCR5 or CXCR3 was detected by a traditional Y2H, in which CXCL12 was fused with the activation domain of GAL4 (AD) as the bait, and CXCR5_NT_ or CXCR3_NT_ was fused with the binding domain (BD) of GAL4 as the preys. Only yeast expressing AD-CXCL12 and BD-CXCR5_NT_ could survive the selective medium, indicating the interaction between prey and bait. (**B**) CoIP of CXCL12 and CXCR5_NT_. CXCL12 and CXCR5_NT_-Fc or CXCR3_NT_-Fc were co-transfected into the 293T cell line. Fc-tagged fusion proteins were precipitated by protein G beads, and the precipitates were separated by SDS-PAGE and blotted with rabbit anti-mouse CXCL12 IgG. The result shows that CXCL12 was precipitated by CXCR5_NT_-Fc, but not by CXCR3_NT_-Fc or Fc only, indicating an interaction between CXCR5_NT_ and CXCL12.

To further verify the interaction between CXCL12 and CXCR5_NT_, we tested the PPIs between CXCL12 and the N-terminus of CXCR5 by a traditional Y2H assay and a co-immunoprecipitation (CoIP) assay (Fig. 5A,B). Both assays indicated an interaction occurs between CXCL12 and CXCR5_NT_.

### General Applicability Test

**Figure 6:**
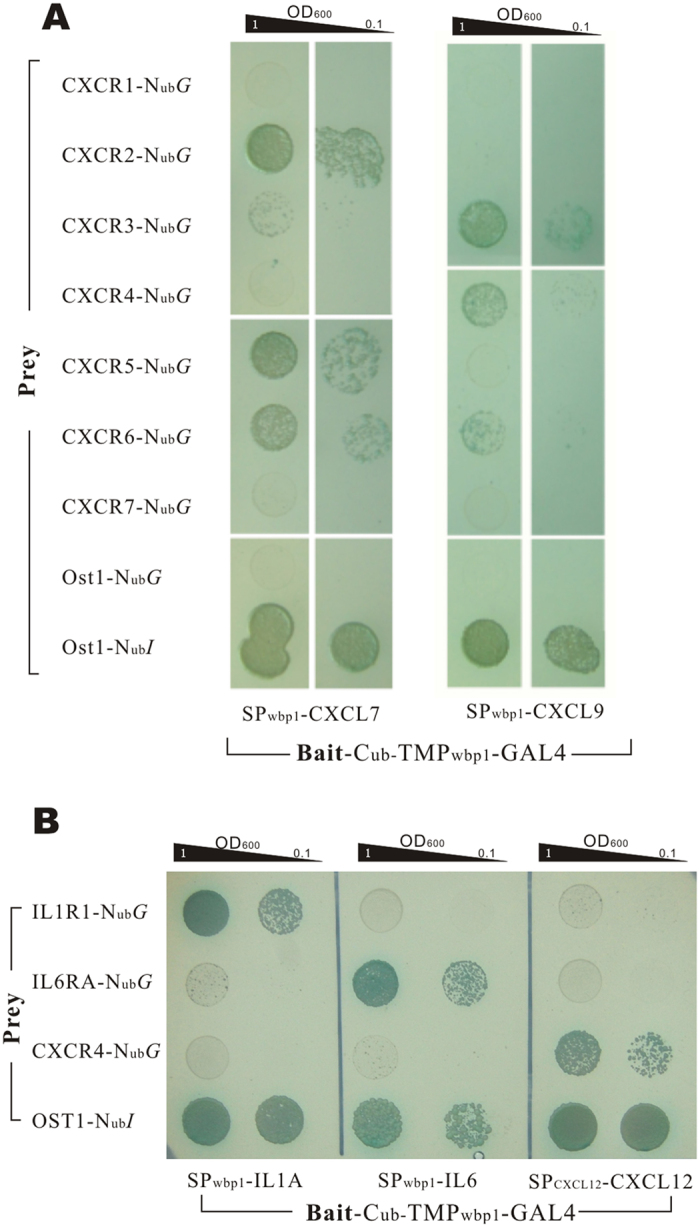
Universality study of the MALAR Y2H method. (**A**) CXCL7 and CXCL9 were detected the PPIs with receptors of CXCR family (CXCRn-N_ub_G) using growth assay, and the result showed different PPIs intensity with each receptors, in which obvious PPIs were detected between CXCL7 and CXCR2 and between CXCL9 and CXCR3. (**B**) Interleukin ligand/receptor pairs, IL1A/IL1R1 and IL6/IL6RA, were detected the interactions. CXCL12 and CXCR4 was used as negative controls of bait and prey respectively, and Ost1-N_ub_*I* was used as positive prey control. Through growth assays, the expected interactions of IL1A/IL1R1 and IL6/IL6RA were detected correctly, meanwhile, no crosstalk was detected between unmatched ligand/receptor pairs.

To test the reliability and robustness of the method, we first examined the PPIs between chemokine ligands CXCL7, CXCL9 and CXCR receptors, which are also members of the CXC chemokine family. In the assays, the ligands were fused with SP_wbp1_ signal peptide (Figure S3A,B). Interactions between CXCL7 and CXCR2, and CXCL9 and CXCR3 were obviously detected by growth assays (Fig. 6A) and β-Gal activity assay (Figure S3C,D). The result was well coincident with the previous reports^10^,^11^,^12^.

Additionally, the PPIs between IL1A/IL1R1 and IL6/IL6RA were also assayed. IL-1A or IL-6 guided by signal peptide of *Wbp1* was detected for their interactions with their receptors respectively (see protein structures in Figure S4A,B). The growth ability assays indicated that the two ligands interacted intensively with their corresponding receptors respectively, while no cross reaction was detected. Meanwhile, CXCL12 and CXCR4, used as negative controls in the assay, did not shown any PPIs with the interleukin ligands or receptors (Fig. 6B) and the same result was also obtained from the β-Gal activity assay (Figure S4C).

## Discussion

Since Y2H technique was first invented, numerous Y2H variants have been designed for different purposes^13^. These Y2H variants have investigated PPIs between cytosolic proteins^13^,^14^, between membrane proteins and cytosolic proteins, and between membrane proteins^4^,^15^,^16^. The powerful high-throughput feature of Y2H has made this technique indispensable in interatomic research of such interactions^3^,^17^.

In the present study, we established a MALAR-Y2H system to detect PPIs between secreted proteins and transmembrane proteins. This MALAR-Y2H system enriches the variety of Y2H variants currently available and extends its application range. The system detects PPIs between secreted proteins anchored on the outer suface of the plasma membrane and native transmembrane proteins, thereby facilitating interactions under native physiological conditions. However, this is often not the case for other *in vitro* assays.

CXCL12/CXCR4 and CXCL12/CXCR7 axes are well-studied chemokine-signaling pathways that are involved in many physical processes^7^,^8^. The interactions between CXCL12 and CXCRs are a good sample for verifying the feasibility of the MALAR-Y2H system. In this system, the key to its success was whether *S. cerevisiae* could recognize the signal peptides and the transmembrane peptides fused with bait and prey proteins. The results proved that yeast, as a lower eukaryotic organism, is compatible with the signal peptide and transmembrane peptides of mammalian genes. Mouse CXCL12, guided by its own signal peptide, can be anchored on the surface of the *S. cerevisiae* plasma membrane as well as the signal peptide of the yeast gene *Wbp1*. In addition, *S. cerevisiae* can recognize the transmembrane peptides of CXCRs and direct their localization to the plasma membrane in the correct orientation. These characteristics, in addition to the simple culture condition and the genetic stability, make yeast a good platform to study PPIs of xenogeneic eukaryotic transmembrane proteins.

Using the presented method, the interactions between chemokines and receptors have been readily assayed. PPIs between CXCL12 and seven CXCRs, and eight chimeric receptor mutants were studied with neither radioactive labels nor protein purification procedures, illustrating the ease and simplicity of the method. The whole experiment was completed within four weeks. Moreover, because the interaction strengths were assayed semi-quantitatively, the system can yield general information that describes the intensity of interactions between CXCL12 and candidate receptors.

Using the Y2H system, high-affinity interactions between CXCL12 and its receptor CXCR4 and CXCR7 were observed, which is in agreement with previous results^18^. What more important, however, is that a complex crosstalk between CXCL12 and other CXC motif receptors was discovered by the Y2H system during the verification. We observed that CXCR5 and CXCR1 also interacted with CXCL12, suggesting that CXCL12 may function as an agonist or antagonist of CXCR1 and CXCR5. These results are in agreement with current opinions concerning the possible abundant crosstalk between CXC chemokines and receptors^19^.

During the following experiments, the advantages of the method was further proved by successfully detecting the PPIs between CXCL7, CXCL9 and their CXC receptors, and between IL1A and IL6 and their receptors, IL-1R1 and IL-6RA^20^,^21^. The former assays confirmed the reliability of the method in detecting the PPIs between ligands and multiple-pass transmembrane receptors once again, while the latter assays demonstrated that the method is also competent to detecting PPIs involving single-pass transmembrane receptor. The single-pass transmembrane proteins are very different from the multiple-pass ones in topological structures, localization mechanisms, interaction patterns with ligands, and so on^6^. These successful assays of the PPIs between variant ligands and receptors make us firmly believe the method possesses a good universality.

At present, the MALAR-Y2H system was only used in lower throughput screening, namely one-to-one matching, as described in this article. However, in most cases, we need to figure out the receptors of a ligand, which has no available candidate receptors for use in such screening. Therefore, we need a system which can do a high throughput screening just as performed in other Y2H systems. Herein, we can propose a solution to upgrade the MALAR-Y2H into a high throughput cDNA library screening system.

**Figure 7:**
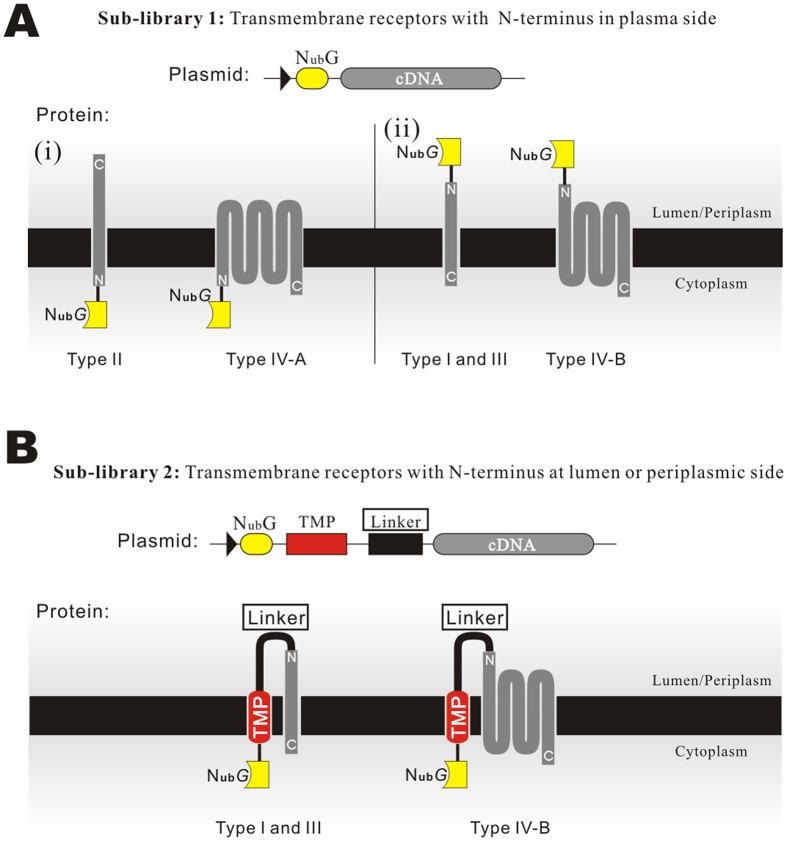
Strategy of the transmembrane protein library construction. (**A**) Type II and IV-A transmembrane proteins have an N-terminus on the inner side of the cytoplasmic membrane. N_ub_*G* is directly tagged at the 5′ end of the cDNA as a report module. (**B**) Type I, III, and IV-B proteins have the N-terminus located on the outside of the cytoplasmic membrane. The N_ub_*G* was fused to the 5′ end of the cDNA by a transmembrane peptide and a linker. The transmembrane peptide mediates the intracellular localization of N_ub_*G*, at the same time, the linker provides the N-terminus of preys sufficient flexibility to maintain their native structures.

Firstly, just as usual, the bait plasmids can be constructed as described above easily. Then, the prey library can be established with the prey plasmid of MALAR-Y2H, though it is a little bit more complicated due to the different topological structures of transmembrane proteins, which must be taken into consideration. On the one hand, the detection module, N_ub_*G*, must be fused with cDNAs at their N-terminus rather than C-terminus (because of the termination codon of the cDNA). On the other hand, the fused N_ub_*G* must be kept into the cytoplasmic side to make sure it able to reconstruct with the C_ub_ on the bait protein once a prey interacts with the bait. Consequently, a single library that simply fuses N_ub_*G* directly to 5′-end of cDNAs is not enough to satisfy the formation of an efficient reporter system for all types of prey proteins. According to topological classification, type II and IV-A transmembrane proteins have the N-terminus located on the inside of the plasma membrane^6^ (Fig. 7A). With regard to these proteins, the directly fused N_ub_*G* motif could be correctly kept on the cytoplasmic side as expected (Fig. 7A-i) and the interaction of baits and preys can unquestionably cause the reconstruction of N_ub_*G* and C_ub_. While, for type I, III, and IV-B proteins, with N-terminus natively locating on the outside of the plasma membrane, the N_ub_*G* will be leaved on the outside surface and the leaved N_ub_G can never contact with the C_ub_ of bait (Fig. 7A-ii) to induce signal cascading.

To resolve the problem, one more cDNA sub-library needs to be generated, in which the total cDNAs are fused at the downstream of the N_ub_G with an additional transmembrane peptide plus a linker between them (Fig. 7B). Through this strategy, the N_ub_*G* that fused with type I, III and IV-B transmembrane proteins will be translocated to the inner surface of the plasma membrane via the transmembrane peptide. It needs to be noted that the linker should be long enough to give the N-terminus of the prey proteins sufficient freedom to maintain their native structures and, at the same time, ensure the linker does not interact with the bait.

By using the above two kinds of sub-libraries individually or in combination, with all types of transmembrane proteins being tagged with the N_ub_*G* module locating on the inside of the plasma membrane, the high throughput screening of PPIs of ligand and transmembrane receptors can be eventually realized. In addition, some prey libraries tagged with N_ub_*G* at the N- or C-terminus are commercially available^22^.

In summary, the present study elaborated how the MALAR Y2H system had been used to study the PPIs between secreted proteins and transmembrane receptors. The reliability and high efficiency were fully revealed and verified. Through the further research, the method could be potentially developed into a high-throughput library screening method.

## Materials and Methods

### Cell lines and Plasmids

*Eerichia. coli* strain DH5*α* was used as the host for plasmid construction and amplification. Yeast strains GoldY2H (MATa, trp1-901, leu2-3, 112, ura3-52, his3-200, gal4Δ, gal80Δ, LYS2:: GAL1_UAS_-Gal1_TATA_-His3, GAL2_UAS_-Gal2_TATA_-Ade2 URA3::MEL1_UAS_-Mel1_TATA_ AUR1-C MEL1) and Y187 (MAT*α*, ura3-52, his3-200, ade2-101, trp1-901, leu2-3, 112, gal4Δ, met–, gal80Δ, URA3::GAL1_UAS_-GAL1_TATA_-lacZ), plasmids pGAD-T7 and pGBK-T7 were purchased from Clonetech, USA. The 293T cell line was maintained by our lab. The pcDNA3.1(−) plasmid was purchased from Life Technology, USA.

### Construction of Bait Plasmids

The bait was expressed by the pGAD-T7 plasmid, in which all elements between the ADH1 promoter and ADH1 terminator were deleted and the target sequences were cloned by a one-step clone kit from Vazyme, Co. China. The bait protein consisted of a signal peptide, a ligand protein, a transmembrane peptide, the C-terminus of yeast ubiquitin (C_ub_), and reporter genes. A signal peptide from one of three genes, SP_CXCL12_ (signal peptide of CXCL12, residues 1–28), SP_WBP1_ (WBP1, residues 1–31) or SP_MFAL1_ (MFAL1, residues 1–89), was fused at the N-terminus of ligand in different experiments. The WBP1 transmembrane peptide and flanking sequences (residues 350–430) were used as the transmembrane peptide and linkers, and fused between CXCL12 and the reporter genes. EGFP was used as a reporter gene to exam the subcellular localization. The C_ub_-GAL4 cassette was used as a reporter module for the detection of PPIs (Fig. 1A).

### Construction of Prey Plasmids

Prey genes were cloned into the pGBK-T7 vector, in which the original ORF between the ADH1 promoter and terminator was deleted, and replaced with mouse CXC motif receptors and reporter genes. EGFP was used as a reporter gene to examine the subcellular localization or N_ub_*G* was used as a reporter module for the detection of PPIs (Fig. 1B).

### Membrane Staining and Co-localization Assay

A lipophilic dye, DiI (Beyotime, China (C1036)), was solved by DMSO to 1 mM. Cells (0.5 ml, OD_600_ = 2.0) were paraformaldehyde fixed on ice for 10 minutes, and washed twice and resuspended with 0.5 ml phosphate buffered solution (PBS), and stained for 10 minutes with 10 μM DiI, and washed twice and mounted with PBS. Fluorescent light of EGFP and Dil (λex: 543 nm, λem: 565 nm) and transmission light were captured by Leica confocal system.

### Yeast Plasmid Transformation and Mating

Plasmids were transformed into yeast cells using the LiAc method, as described in the commercial kit manual. Bait plasmids were transformed into GoldY2H cells, whereas prey plasmids were transformed into Y187 cells. Transformants were selected with synthetic dropout nutrient medium/plate (SD medium) without leucine or tryptophan (SD/Leu^−^ or SD/Trp^−^). Mating was performed by suspending 2–3 clones of each cell into 0.5 ml YPD medium, and incubating overnight with shaking. Cells were collected by centrifugation and spread onto SD/Leu^−^Trp^−^ media.

### Predicting Transmembrane Topological Structures

The transmembrane topological structure of all fusion transmembrane proteins was predicted by an online tool: (http://www.cbs.dtu.dk/services/TMHMM/, a hidden Markov model for predicting transmembrane helices in protein sequences).

### Self-Activating Assay

Self-activation of bait proteins was detected by testing cell growth and a color shift after culturing cell suspensions on SD/Leu^−^His^−^Ade^−^ media with or without 3-AT.

### Interaction Strength Detection

#### Growth assay

Five microliters of 10-fold diluted cells suspension, OD_600_ = 1, 0.1 and 0.01 series, were dropped on SD/Leu^−^Trp^−^His^−^Ade^−^ medium plates. Cell growth was recorded by photographing the plates after 72 h incubation.

#### β-Galactosidase (β-Gal) Activity Assay

Cells were grown in liquid SD/Leu^−^Trp^−^ medium to an OD_600_ of 0.6–0.8. A one milliliter culture was harvested by centrifugation and suspended in 100 μl Z buffer (60 mM Na_2_HPO_4_, 40 mM NaH_2_PO_4_, 10 mM KCl, 1 mM MgSO_4_, pH 7.0). Cells were lysed through three freezing and thawing cycles in liquid nitrogen and a 37 °C water bath. Seven-hundred microliters of Z buffer containing 0.27% (v/v) 2-mercaptoethanol and 160 μl Z buffer containing 0.4% 2-nitrophenyl-*β*-D-galactopyranoside were added and incubated for 30–60 min at 30 °C. Four hundred microliters of 1 M Na_2_CO_3_ was added to stop the reaction. The sample was centrifuged and the supernatant absorbance was measured at 420 nm. β-Gal activity was calculated by the equation (1).


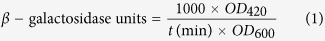


#### Western Blot

Cells were grown in 1 ml liquid SD/Leu^−^Trp^−^ medium at 30 °C overnight to an OD_600_ of 0.6–1.0. Proteins were extracted according to a modified post-alkaline extraction method^23^. Briefly, 2 ml culture of yeast cells (OD_600_ of 0.6–1.0) were harvested by centrifugation and resuspended in 100 μl distilled water and 100 μl of 0.2 M NaOH was added. This solution was incubated for 5 min at room temperature. Cells were pelleted by centrifugation and resuspended in 50 μl SDS-PAGE sample buffer and incubated at 95 °C in a water bath for 5 min. Samples were separated by SDS-PAGE and transferred to a PVDF membrane. GAL4 was detected by a rabbit anti-GAL4 IgG (abcam, ab1396), and CXCL12 by a rabbit anti-mouse CXCL12 IgG (abcam, 9797). The Fc-tagged CXCR5_NT_ and CXCR3_NT_ were detected by a HRP-labeled donkey anti-human IgG.

### Construction of Chimeric Receptors

Extracellular fragments of CXCR5 and CXCR3 were determined according to the UniProt database. The sequences are shown in Figure S2. Insert fragments were cloned seamlessly into recipient genes using the one-step clone kit from Vazyme, Co. China (C112-01). All plasmids were confirmed by sequencing.

### CoIP of CXCL12 and CXCR5_NT_

The N-terminal fragment of the CXCR5 fragment (residues 1–55) was fused with the human IgG1 Fc fragment (CXCR5_NT_-Fc) and inserted into vector pcDNA3.1(−). The CXCL12 mature protein CDS was cloned into pcDNA3.1(−). Plasmids were co-transfected into the 293T cell line, and lysed at 48 h for CoIP. Co-transfection of CXCR3_NT_ (1–52)-Fc and CXCL12 was used as the negative control.

Fifty microliter protein A-coupled Sepharose beads were blocked by PBS containing 0.1% BSA and incubated with cell lysis buffer at 4 °C for 4 h, and washed with the lysis buffer three times. The beads were suspended with 50 μl 2×SDS-PAGE loading buffer and boiled for 5 min. Samples were separated by SDS-PAGE and blotted to PVDF membranes.

### Classic Intracellular Y2H of CXCL12 and CXCR5_NT_

Mature CXCL12 was fused to the activation domain of GAL4 in the pGAD-T7 plasmid, and CXCR5_NT_ or CXCR3_NT_ was fused to the binding domain of GAL4 in the pGBK-T7 plasmid, according to the instructions from the manufacturer. Transformation, mating and detection were performed according to the above method [Yeast Plasmid Transformation and Mating].

## Additional Information

**How to cite this article**: Li, J. *et al*. Development of a membrane-anchored ligand and receptor yeast two-hybrid system for ligand-receptor interaction identification. *Sci. Rep.* **6**, 35631; doi: 10.1038/srep35631 (2016).

## Supplementary Material

Supplementary Information
