# Supplementary material for: Development of a membrane-anchored ligand and receptor yeast two-hybrid system for ligand-receptor interaction identification
Source: Sci Rep. 2016 Oct 20;6:35631. doi: 10.1038/srep35631 (PMC5071910; doi:10.1038/srep35631)
Supplement: Supplementary Information [file srep35631-s1.pdf]

Supplemental Information

Development of a membrane-anchored ligand and receptor yeast two-hybrid system for ligand-receptor interaction identification

Jingjing Li, Jin Gao, Lei Han, Yinjie Zhang, Wen Guan, Liang Zhou, Yan Yu, and Wei Han

Contents: 4 figures (Fig. S1, S2, S3, S4)

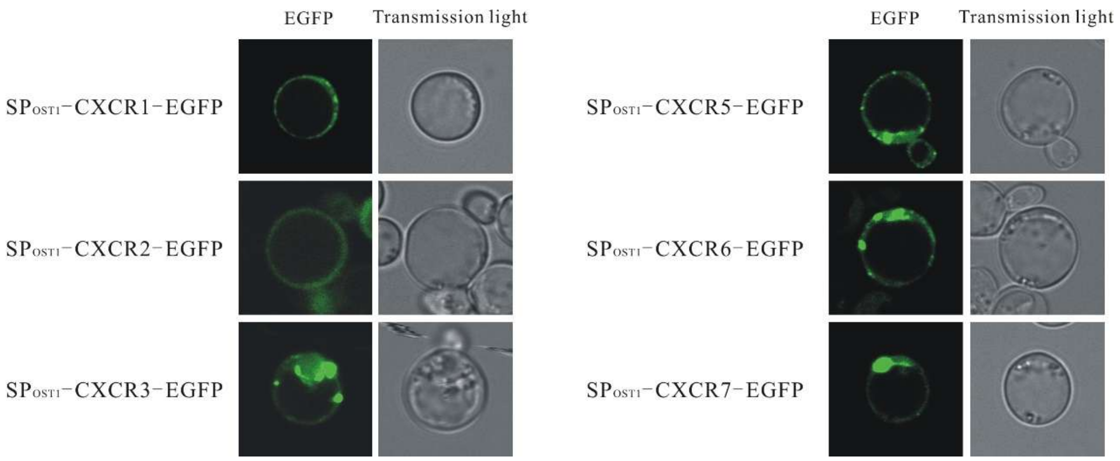

Fig. S1 The subcellular localization of CXC receptors. CXC receptors were directed by signal peptide of S. cerevisiae OST1 gene and expressed in yeast GoldY2H strain. The EGFP fluorescence was observed by laser scanning confocal microscope. The result indicates these receptors were well directed on cytoplasmic membrane.

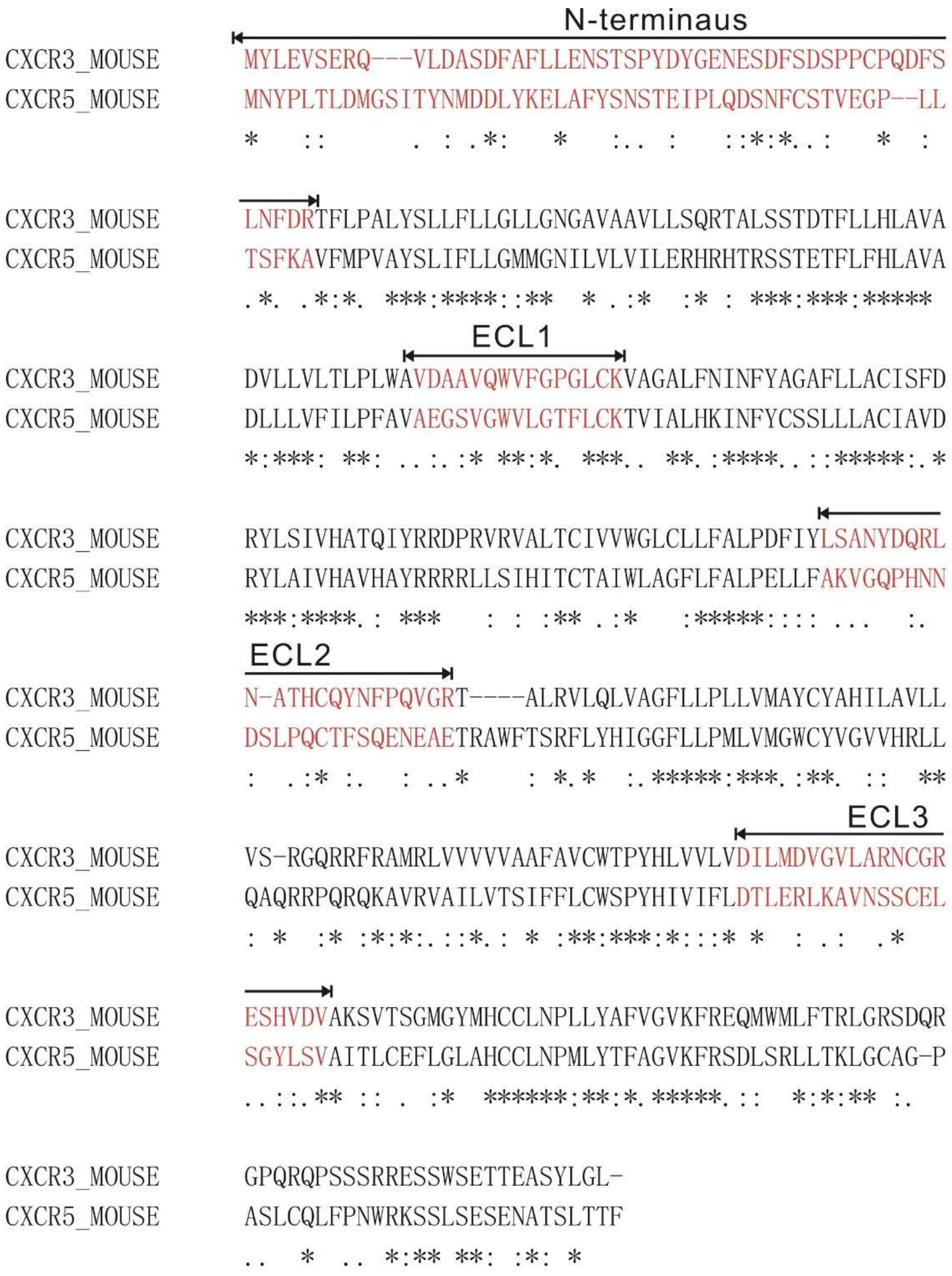

Fig. S2 The extracellular domain partitions of CXCR3 and CXCR5. Partitions were performed according to UniProt database (http://www.uniprot.org/), and ClustalX1.8 software. Red letters denote the extracellular domains.

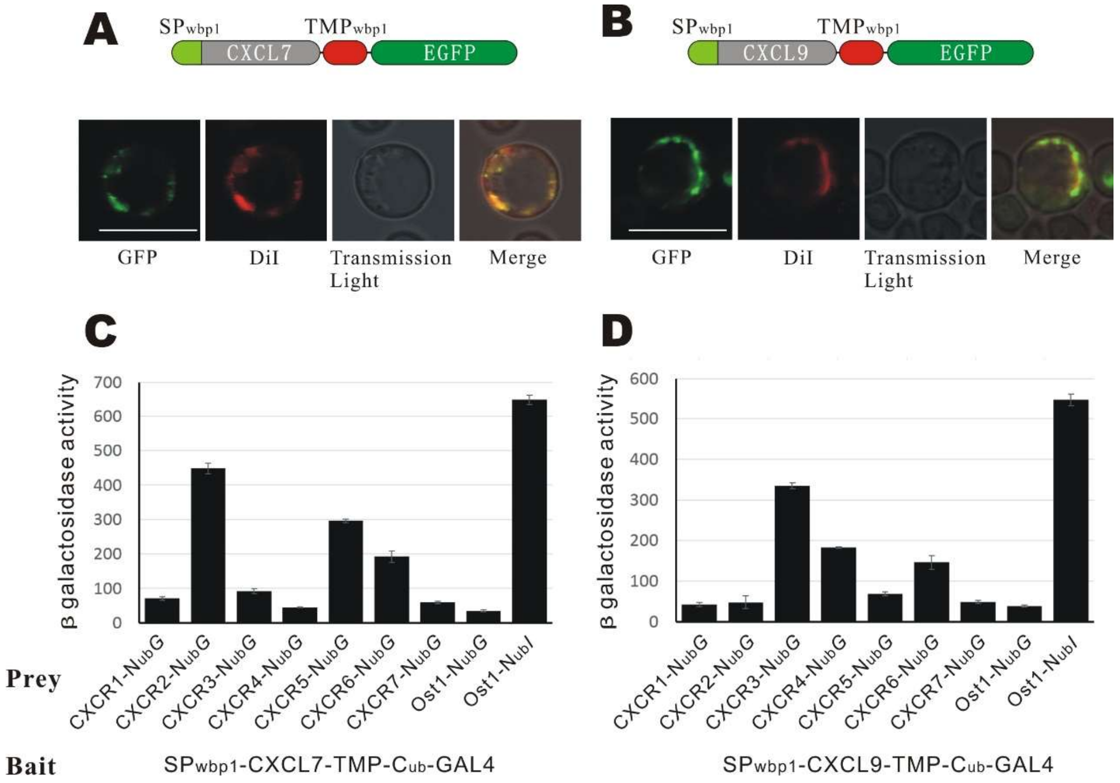

Fig. S3. PPIs detection between CXCL7 and CXCL9 and CXCR family. (A) Mouse *Cxcl7* and **(B)** *Cxcl9* mature peptide encoding sequences were fused with *Wbp1* signal peptide (SP_{wbp1}) at the N-terminus and *Wbp1* transmembrane peptide at the C-terminus. Their subcellular localization was reported by an EGFP tag at the C-terminus. The EGFP fluorescence was well colocalized with the DiI red fluorescence, indicating the bait proteins localized on plasma membrane. **(C-D)** The β-galactosidase activity assay was performed and the results coincident with that of growth assay (Fig. 6A). In the above experiment, prey Ost1-N_{ub}*G* was used as the negative control and Osti-N_{ub}*I* as positive control.

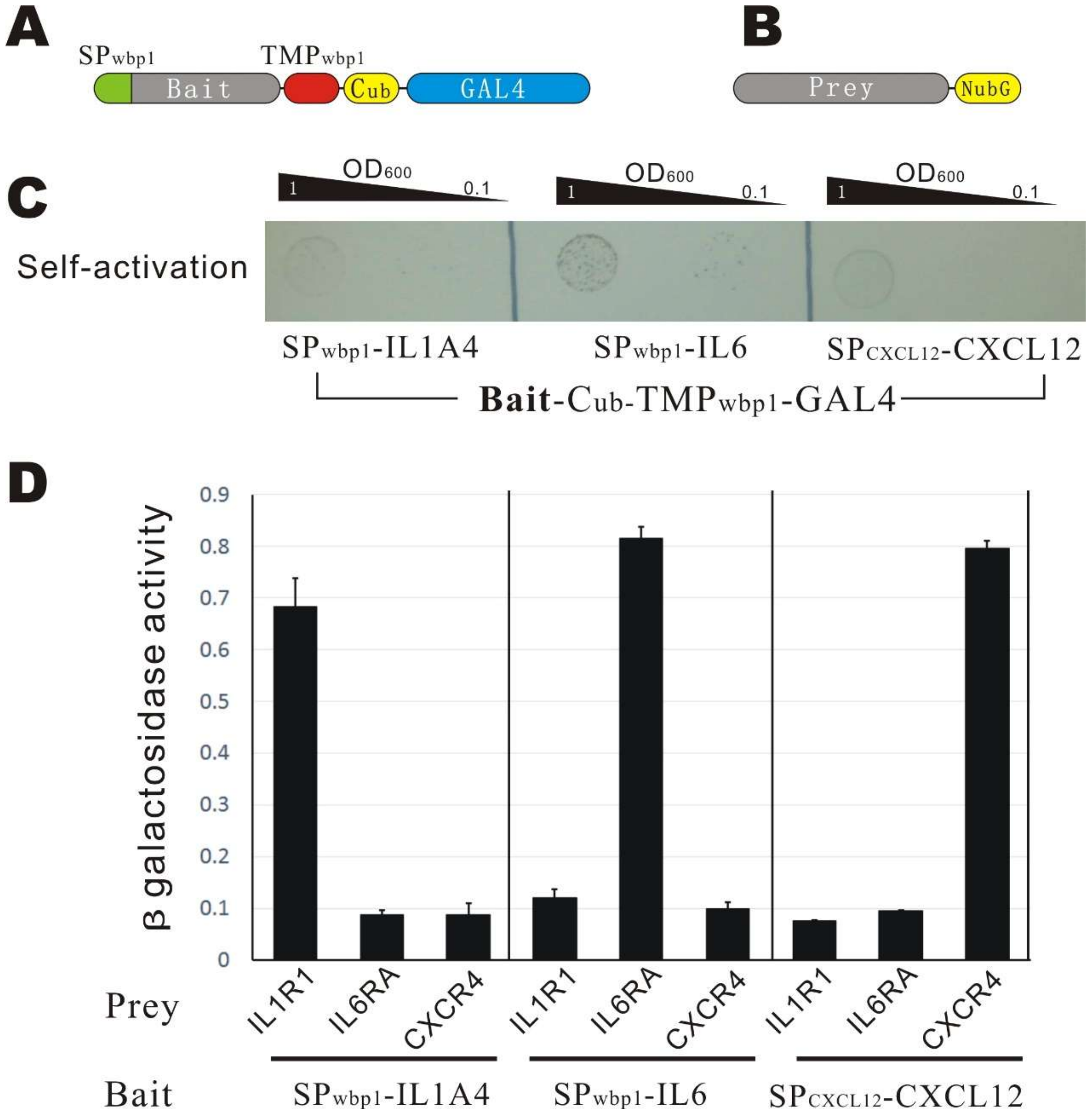

Fig S4. PPIs detection between IL1A and IL6 and IL1R1 and IL6RA. (A) Mouse IL1A or IL6 mature peptide encoding sequence were fused with *Wbp1* signal peptide (SP_{wbp1}) at the N-terminus and *Wbp1* transmembrane peptide at the C-terminus. **(B)** Prey plasmids were constructed by fusing receptor genes (*IL1R1* or *IL6RA*) with $N_{ub}G$. **(C)** The self-activation of the baits were detected, but no obvious activation was observed. **(D)** The interactions of baits and preys were evaluated by β-galactosidase activity assay. The results were consistent with that of growth assay in Fig 6B.
